# IL17A Depletion Affects the Metabolism of Macrophages Treated with Gemcitabine

**DOI:** 10.3390/antiox10030422

**Published:** 2021-03-10

**Authors:** Cecilia Roux, Gianluca Mucciolo, Joanna Kopecka, Francesco Novelli, Chiara Riganti, Paola Cappello

**Affiliations:** 1 Center for Experimental Research and Medical Studies (CERMS), AOU Città della Salute e della Scienza di Torino, University of Turin, 10126 Turin, Italy; cecilia.roux@unito.it (C.R.); gianluca.mucciolo@unito.it (G.M.); franco.novelli@unito.it (F.N.); 2 Department of Molecular Biotechnology and Health Sciences, University of Turin, 10126 Turin, Italy; 3 Department of Oncology, University of Turin, 10126 Turin, Italy; joanna.kopecka@unito.it (J.K.); chiara.riganti@unito.it (C.R.); 4 Molecular Biotechnology Center, via Nizza 52, 10126 Turin, Italy

**Keywords:** Interleukin 17A (IL17A), macrophages, metabolism, pancreatic cancer, chemo-immunotherapy

## Abstract

Background: Interleukin (IL)17A is a member of the IL17 cytokine family, which is released by both immune and non-immune cells such as tumor and stromal cells into the tumor microenvironment. IL17 receptors are also widely expressed in different type of cells. Among all the members, IL17A is the most controversial in regulating tumor immunity. Here, we investigated how IL17A inhibition modulated macrophage differentiation and metabolism in the presence or absence of gemcitabine. Gemcitabine is the gold standard drug for treating pancreatic cancer and can increase macrophage antitumoral activities. Results: We observed some unique features of macrophages polarized in the absence of IL17A, in terms of RNA and protein expression of typical phenotypic markers, and we demonstrated that this paralleled specific changes in their metabolism and functions, such as the induction of an antitumor response. Interestingly, these features were almost maintained or enhanced when macrophages were treated with gemcitabine. We also demonstrated that the anti-IL17A antibody effectively reproduced features of macrophages derived from IL17A knock-out mice. Conclusion: Overall, we provide a proof-of-concept that combining an anti-IL17A antibody with gemcitabine may represent an effective strategy to modulate macrophages and enhance the anti-tumor response, especially in pancreatic cancer where gemcitabine is widely used.

## 1. Introduction

While T helper (Th)17 cells play a well-established role in inflammation and autoimmune diseases, their role in tumor immunity remains controversial. Th17 cells have been implicated in protective immunity and disease outcome, as well as in the amplification of the inflammatory response that favors tumor initiation, progression and resistance [1] and allergic diseases, especially asthma [2]. Interleukin (IL)17A is the founding member of the IL-17 family, which has five other members, designated IL-17A–F. IL17A can either exist as homodimers or as IL17A-IL17F heterodimers, which bind IL17 receptor A and C (RA/RC) heterodimer receptors [3]. Both receptors are present on many immune and non-immune cells, including tumor epithelial cells such as transformed acinar and ductal pancreatic cells [4].

IL17A plays a role in neutrophils, but has also been implicated in the activation of different immune cells including macrophages. Macrophages are crucial players in inflamed tissues as well as in tumors where, based on their cytokine-dependent differentiation status, can limit or support tumor evasion from immunosurveillance. A well-accepted dichotomous classification defines macrophages (especially when differentiated in vitro) as M1, which are “classically activated” (when stimulated by Toll Like Receptor (TLR) ligands and Interferon (IFN)γ), or M2, which are “alternatively activated” (when stimulated by Interleukin (IL)4/IL13) [5,6]. M1 macrophages are also regarded as inflammatory due to the high production of pro-inflammatory cytokines, reactive nitrogen and oxygen species (ROS) and strong tumoricidal activity. Conversely, M2 macrophages are considered immunosuppressive due to their immunoregulatory functions; they mainly foster tissue remodeling and tumor progression [7].

The effect of IL17A on the macrophage population is not fully known, probably because different macrophage subpopulations modulate IL17R expression in response to several inflammatory stimuli [8]. Moreover, most published studies report an in vivo effect of IL17A on macrophages isolated from inflamed tissues. Erbel and colleagues observed that IL17A caused atherosclerotic macrophages to assume an M1-like phenotype [9], while Nishikawa et al. described the central role of IL17A in inducing an M2-like population to regulate intestinal inflammation [10]. We previously demonstrated that IL17A, released by macrophages after treatment with taxol, contributes to render them more suppressive [11].

In addition to the phenotypical features, M1 and M2 macrophages display differences in many metabolic pathways. The current classification defines M1 macrophages as characterized by an activated glycolytic pathway, pentose phosphate pathway (PPP) and fatty acid synthesis (FAS), while M2 macrophages are characterized by activated fatty acid oxidation (FAO) and oxidative phosphorylation (OXPHOS) [12]. However, recent studies have demonstrated that glycolysis is induced in both M1 and M2 macrophages, but only M1 macrophages also have increased PPP [13], and FAO is still controversially involved in both M1 and M2 macrophages [12]. Here, we report that IL17A neutralization was shown to modulate in vitro macrophage polarization per se, both in terms of metabolism activation and of RNA and protein expression of typical markers. In particular, IL17A neutralization opposed IL4 in driving the M2 phenotype and metabolic polarization, but some features typical of M2 macrophages were maintained, together with those associated with M1 macrophages, suggesting the acquisition of a unique phenotype. We also report that these features still remained in the presence of gemcitabine, a chemotherapy drug widely used in pancreatic cancer treatment (alone or in combination with other drugs), whose effects on macrophages are under debate [14,15]. In addition, it has been already proven to indirectly influence macrophages in vitro and in vivo [15,16].

In conclusion, we demonstrate that IL17A neutralization represents a novel strategy to shape tumor-associated macrophages and might potentiate the gemcitabine effect on these cells. 

## 2. Materials and Methods

### 2.1. In Vitro Generation of Murine Bone Marrow-Derived Macrophages (BMDM)

Whole bone marrow was harvested from 10- to 12-week-old female mice by flushing RPMI medium through femurs and tibias using a 27-gauge needle. Animal use for bone marrow collection has been authorized by the Italian Ministry of Health, Animal and Veterinary Office (authorization No. 597/2019-PR). Following red blood cell lysis, cells were cultured overnight in 10% RPMI in 10-cm plates. Non-adherent cells were collected and seeded in Petri dishes in medium containing 20 ng/mL macrophage colony-stimulating factor (M-CSF) (PeproTech, supplied by Tebu-Bio S.r.l., Magenta MI, Italy). After 3 days of culture, cell media were replenished with fresh medium and M-CSF. BMDM were harvested on day 4, and used for all in vitro assays. Polarization was obtained by culturing BMDM with medium containing IL4 (20 ng/mL; PeproTech) and M-CSF (20 ng/mL; PeproTech), or 100 ng/mL LPS (PeproTech) for 24 h. Hereinafter, IL4+ M-CSF-stimulated macrophages will be referred to as M2-like, and LPS-stimulated macrophages as M1-like cells. When specifically indicated, gemcitabine (1 mg/mL), or anti-IL17A antibody (20 μg/mL; BE0173, BioXCell, supplied by D.B.A. Italia S.r.l., Segrate MI, Italy) or its isotype control (BE0083, BioXCell), or recombinant IL17A (PeproTech) were added parallelly to the polarizing stimuli for 24 h. If not differently specified, all metabolic and molecular biology assays were performed at 24 h after treatment.

### 2.2. Quantitative RT-PCR (qPCR)

RNA was isolated using the Nucleospin RNA Plus kit (Macherey-Nagel, supplied by Carlo Erba Reagents S.r.l., Cornaredo MI, Italy), and reverse transcribed using the iScript cDNA Synthesis kit (Bio-Rad, Segrate MI, Italy) according to the manufacturer’s instructions. qPCR was performed using SYBR Green primers (Applied Biosystems, supplied by Thermo Fisher Scientific, Monza, Italy). Mouse glyceraldehyde 3-phosphate dehydrogenase (gapdh) was used as a housekeeping gene; the relative mRNA expression was calculated using M0 cells, each time, as controls to compare the effect of genotype in polarization, and each untreated M1 or M2 to compare the effect of gemcitabine on the polarization of both wild-type (WT) and IL17A^−/−^ cells. All primer sequences are listed in Table 1. 

### 2.3. ELISA

Supernatants from differently stimulated BMDM were assessed for the presence of IFNγ (eBioscience 88-7314-88, supplied by Thermo Fisher Scientific, Monza, Italy), IL10 (R&D Systems, supplied by Bio-Techne, Milan, Italy), IL6 (Biolegend, supplied by Campoverde S.r.l., Milan, Italy) and TNFa (R&D Systems) through an Enzymatic-Linked Immunosorbent Assay (ELISA) following the manufacturer’s instructions. Optical Density values were measured at 450 nm with the VICTOR Nivo Multimode Microplate Reader (PerkinElmer, Milan, Italy).

### 2.4. MTT Assay

Cell viability was assessed by the MTT assay. Cells were seeded in 96-well plates at 1.5 × 10^3^ cells/well, and 20 μL of 5 mg/mL MTT solution (Sigma-Aldrich, Milan, Italy) was added to each well, and incubated at 37 °C for a further 4 h every 24 h. Plates were centrifuged for 10 min at room temperature and medium was removed. The insoluble formazan product was dissolved in 200 μL of dimethyl sulfoxide (Sigma-Aldrich) for 10 min at room temperature on a platform shaker. Optical Density values were measured at 570 nm with the VICTOR Nivo Multimode Microplate Reader (PerkinElmer).

### 2.5. The Pentose Phosphate Pathway (PPP) and Tricarboxylic Acid (TCA) Cycle

After washing in PBS, cells were detached with PBS containing 2.5% *v*/*v* FBS and 2 mmol/L EDTA, rinsed with PBS, and resuspended in 1 mL Hepes buffer (145 mmol/L NaCl, 5 mmol/L KCl, 1 mmol/L MgSO_4_, 10 mmol/L Hepes, 10 mmol/L glucose, 1 mmol/L CaCl_2_, pH 7.4). 50 μL were taken up, sonicated and used to measure the protein content. In each remaining sample, 2 μCi of [6-^14^C] glucose (55 mCi/mmol, PerkinElmer) or 2 μCi of [1-^14^C] glucose (58 mCi/mmol, PerkinElmer) were added. The labelled cell suspension was incubated for 1 h in a closed tube to trap the ^14^CO_2_ produced from the [^14^C] glucose. After this incubation time, the metabolic flux was interrupted by adding 0.5 mL 0.8 N HClO_4_ [17]. [1-^14^C] glucose metabolized through PPP or TCA and [6-^14^C] glucose metabolized through the TCE only produced ^14^CO_2_. The amount of glucose producing CO_2_ through the PPP was obtained by subtracting the amount of [6-^14^C] glucose (TCA cycle) from [1-^14^C] glucose (TCA + PPP cycles), as described [17]. Results were expressed as nmol CO_2_/h/mg cell proteins.

### 2.6. Lactate

The determination of the lactate levels in the cell culture medium was performed with the l-Lactate Assay Kit (Abcam, supplied by Prodotti Gianni S.p.a., Milan, Italy), according to the manufacturer’s instructions. Results were expressed as nmol/mL.

### 2.7. Fatty Acid β-Oxidation

Cells were rinsed twice with PBS, detached with 0.05/0.02% *v*/*v* trypsin/EDTA and centrifuged at 13,000× *g* for 5 min. Protein quantification was performed on 50 μL of the sample after sonication. The remaining samples were re-suspended in culture medium containing 0.24 mmol/L fatty acid-free bovine serum albumin (BSA), 0.5 mmol/L l-carnitine, 20 mmol/L Hepes, 2 μCi [1-^14^C] palmitic acid (3.3 mCi/mmol, PerkinElmer) [18] and transferred into tightly closed tubes. For each experiment, a negative control was performed on cells incubated with the carnitine palmitoyltransferase inhibitor etomoxir (1 μmol/L) for 30 min, a positive control was performed on cells incubated with the AMP-kinase activator 5-aminoimidazole-4-carboxamide ribonucleotide AICAR (1 mmol/L) for 30 min. Samples were incubated 2 h at 37 °C, 1:1 *v*/*v* phenylethylamine/methanol (0.3 mL) and 0.8 N HClO_4_. (0.3 mL) were added. Samples were incubated for an additional 1 h at room temperature, then centrifuged at 13,000× *g* for 10 min. The supernatants, containing ^14^CO_2_, and the precipitates, containing ^14^C-acid soluble metabolites (ASM), were subjected to liquid scintillation count. Results were expressed as pmol of [^14^CO_2_] or ^14^C-ASM/h/mg cell proteins. 

### 2.8. Mitochondrial Respiratory Chain Measurement

Cells were rinsed twice in ice-cold PBS, lysed with 0.5 mL buffer A (50 mmol/L Tris, 100 mmol/L KCl, 5 mmol/L MgCl_2_, 1.8 mmol/L ATP, 1 mmol/L EDTA, pH 7.2) containing the protease inhibitor cocktail III [100 mmol/L AEBSF, 80 mmol/L aprotinin, 5 mmol/L bestatin, 1.5 mmol/L E-64, 2 mmol/L leupeptin and 1 mmol/L pepstatin (MerckMillipore, Milan, Italy) 1 mmol/L PMSF, 250 mmol/L NaF]. Samples were centrifuged at 650× *g* for 3 min at 4 °C, supernatants were transferred into a new tube series and centrifuged at 13,000× *g* for 5 min at 4 °C. The supernatants were discarded, the pellets containing mitochondria, after a washing step with 0.5 mL buffer A, were re-suspended in 0.25 mL buffer B (250 mmol/L sucrose, 15 μmol/L K_2_HPO_4_, 2 mmol/L MgCl_2_, 0.5 mmol/L EDTA, 5% *w/v* BSA). 50 μL were sonicated and used for protein quantification. The activity of mitochondria respiration complexes was evaluated according to [18]. Results were expressed as nmol red cit c/min/mg mitochondrial proteins.

### 2.9. ATP Detection

ATP levels in mitochondrial extracts were evaluated with the ATP Bioluminescent Assay Kit (Sigma-Aldrich), using a Synergy HT Multi-Mode Microplate Reader (Bio-Tek Instruments, supplied by Thermo Fisher Scientific, Monza, Italy). Results as relative light units (RLU) were expressed as nmol ATP/mg mitochondrial proteins, according to the titration curve previously prepared.

### 2.10. Glutamine Catabolism

Glutamine catabolism was measured as described in [19], with minor modifications. Cells were rinsed with PBS, detached, centrifuged at 13,000× *g* for 5 min at 4 °C, re-suspended in 250 μL of buffer A (150 mmol/L KH_2_PO_4_, 63 mmol/L Tris/HCl, 0.25 mmol/L EDTA; pH 8.6) and sonicated to measure the protein content. In the first sample series, 100 μL of each lysate were incubated at 37 °C for 30 min in 850 μL of buffer B (80 mmol/L Tris/HCl, 20 mmol/L NAD^+^, 20 mmol/L ADP, 3% *v*/*v* H_2_O_2_; pH 9.4) and 50 μL of 20 mmol/L l-glutamine. NADH absorbance at 340 nm was followed with a Lambda 3 spectrophotometer (PerkinElmer) and was linear during the whole assay. Results, expressed as μmol NADH/min/mg cell proteins, corresponded to the activity of glutaminase (GLS) plus l-glutamic dehydrogenase. In the second sample series, 20 μL of the GLS inhibitor bis-2-(5-phenylacetamido-1,3,4-thiadiazol-2-yl)ethyl sulfide BTPES (30 μmol/L, i.e., a concentration that inhibited GLS activity at 100%, data not shown), was added after 15 min and NADH absorbance was followed for 15 min. Results, expressed as μmol NADH/min/mg cell proteins, indicated the l-glutamic dehydrogenase activity. GLS activity was obtained by subtracting the rate of NADH production in second assay from the rate of the first assay.

### 2.11. Formalin-Fixed and Paraffin-Embedded (FFPE) Dissociation and Flow Cytometry Analysis

To analyze tumor-associated macrophages (TAMs) and tumor-infiltrating immune lymphocytes from orthotopic PDAC arose in IL17A knock out mice or in wild-type mice treated or not with an anti-IL17A mAb, two 50 μm sections from a FFPE block were transferred into a gentle MACS C tube to be dissociated while preserving intact cells with the FFPE Tissue Dissociation Kit (130-118-052, Miltenyi Biotec, Bologna, Italy) following the manufacturer’s instructions. Five mice per group were analysed. Single cell suspensions were resuspended in PBS containing 0.5% BSA and 0.01% NaN_3_, and unspecific binding sites were blocked by 10 min incubation with anti-CD16/CD32 mAb (BioLegend, clone 93). Staining was performed using the following antibodies: FITC anti-CD11b (Miltenyi Biotec, clone M1/70.15.11.5), PerCP anti-CD45 (Miltenyi Biotec, clone 30F11), FITC anti-CD4 (Miltenyi Biotec, clone REA604), Per-CP anti-CD8 (eBioscience, clone 53-6.7), AF647anti-Granzyme-B (51-8898-82 eBioscience, clone NGZB), APC anti-CD206 (141712 BioLegend, clone C068C2), APC anti-FoxP3 (17-5773-82 eBioscience, clone FJK-16s) and APC anti-IFNγ (17-7311-82 eBioscience, clone XMG1.2). Cells were washed again with 0.5% BSA plus 0.01% NaN_3_ in PBS. For IFNγ, Granzyme-B and FoxP3 staining, cells were stained for surface markers, then fixed and permeabilized at 4 °C for 30 min with the Fixation Permeabilization kit (eBioscience) after several washes with 0.5% BSA plus 0.01% NaN_3_ in PBS. After washing with permeabilization buffer, antibodies against IFNγ, Granzyme-B and FoxP3 were added for 20 min at 4 °C. All flow cytometry data were acquired using the Accuri™ C6 cytometer (BD Biosciences, Milan, Italy) and analyzed with FlowJo_vX.0.7 software (Tree Star from BD Biosciences).

### 2.12. ROS Measurement

To measure intracellular ROS, 2 × 10^5^ BMDM were incubated with either 300 nM DCF-DA (C6827, Invitrogen, supplied by Thermo Fisher Scientific, Monza, Italy) or 5 μM MitoSOX (M36008, Invitrogen) for 10 min at 37 °C. Fluorescence signals were acquired with an Accuri™ C6 cytometer (BD Bioscience), and data were analyzed using FlowJo_vX.0.7 software (BD Bioscience).

### 2.13. Macrophage Uptake Activity

BMDM were seeded onto glass coverslips in 12-well plates (0.5 × 10^5^ cells per well). Cells were co-cultured with carboxyfluorescein succinimidyl ester (CFSE)-labelled (V12883, Invitrogen) K8484 cells pretreated with gemcitabine (1 mM for 24 h), for 2 h, 18 h or 48 h. Cells were incubated with blocking buffer containing anti-CD16/32 Ab in 0.5% BSA in PBS at 4 °C for 10 min, and then incubated with the primary antibody anti-I-A/E^b^ (107608 BioLegend; clone M5/114.15.2) at 0.2 μg/mL for a further 30 min. Cells were fixed with paraformaldehyde (2% PFA) at room temperature for 10 min, and then washed twice with PBS. Cells were then permeabilized with 0.05% Triton-X in PBS at room temperature for 10 min and stained with TO-Pro3 (T3605, Invitrogen)—a nucleophile dye—at room temperature for 30 min. Coverslips were washed twice in 0.05% Triton-X in PBS and mounted with VectaShield (20 μL/coverslip; Vector Laboratories, supplied by D.B.A Italia S.r.l., Segrate MI, Italy). Images were acquired with a confocal microscope (sp8, Leica, Milan, Italy) using LAS X software v.3.4.2 (Leica), and processed with Adobe Photoshop CS5. Data were reported as percentage of cells with CFSE positivity inside per total number of cells. MHC class II staining was used as a control.

### 2.14. Immunoblotting

Untreated mouse WT and IL17A-depleted M1- and M2-like macrophages, or those treated with gemcitabine, were collected and resuspended in lysis buffer (TBS, 0.5% Triton X-100, 0.5% NP-40, 1mM PMSF, 1mM Na_3_VO_4_, 1% DTT and protease inhibitor cocktail). Protein concentration was evaluated using the Bio-Rad Protein Assay (Bio-Rad). Samples were resuspended in 4× Bolt LDS Sample Buffer and 10× reducing agent, and incubated at 70 °C for 10 min before loading on precast Bolt 4–12% Bis-Tris Plus gels (all from Thermo Fisher Scientific, Monza, Italy). Immunoblotting was performed with the following primary Abs: anti-NOS2 (Cell Signaling, supplied by Euroclone, Pero Mi, Italy), anti-ARG1 (Cell Signaling) and anti-HSP90 (Cell Signaling) following the manufacturer’s instructions. Anti–rabbit HRP-conjugated secondary Abs (diluted 1:10,000) were obtained from Thermo Fisher Scientific. Membranes were developed for chemiluminescent detection, and images were acquired using a ChemiDoc MP Imaging System and Image Lab 6.0.1 Software (Bio-Rad). Immunoblots were quantified by ImageJ 1.45 software (National Institutes of Health, Bethesda, MD, USA).

### 2.15. Statistical Analyses

Data in bar graphs are reported as means ± SEM, with *p* values calculated using the Student’s *t* test (* *p* ≤ 0.05, ** *p* ≤ 0.01, *** *p* ≤ 0.001). Means were calculated based on a minimum of *n* = 3 replicates in each independent experiment. Data were analyzed either by Microsoft Excel (Microsoft, Redmond, WA, USA) or GraphPad Prism 8 (GraphPad, San Diego, CA, USA).

## 3. Results

### 3.1. The Absence of IL17A Induces Unique Features in Both M1- and M2-Like Macrophages

**Figure 1:**
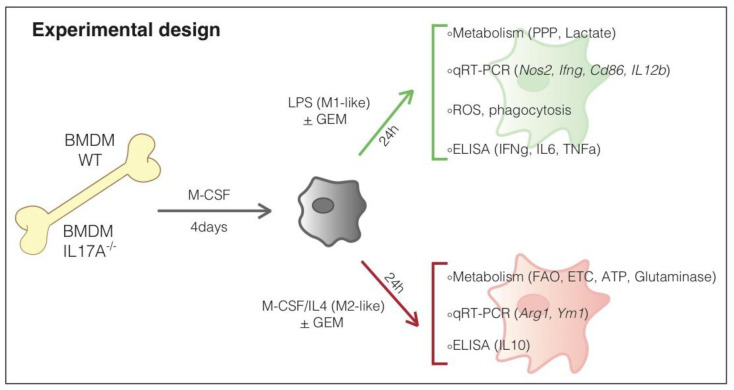
Experimental design.

We firstly evaluated if the absence of IL17A affected the ability of macrophages to polarize in vitro in dichotomic M1- and M2-like cells. BMDM derived from WT or IL17A^−/−^ mice were stimulated for 24 h with LPS, to induce an inflammatory phenotype (M1-like), or with M-CSF + IL4, to induce an anti-inflammatory phenotype (M2-like). mRNA, supernatants and whole cells were collected to be analyzed as listed in Figure 1.

**Figure 2:**
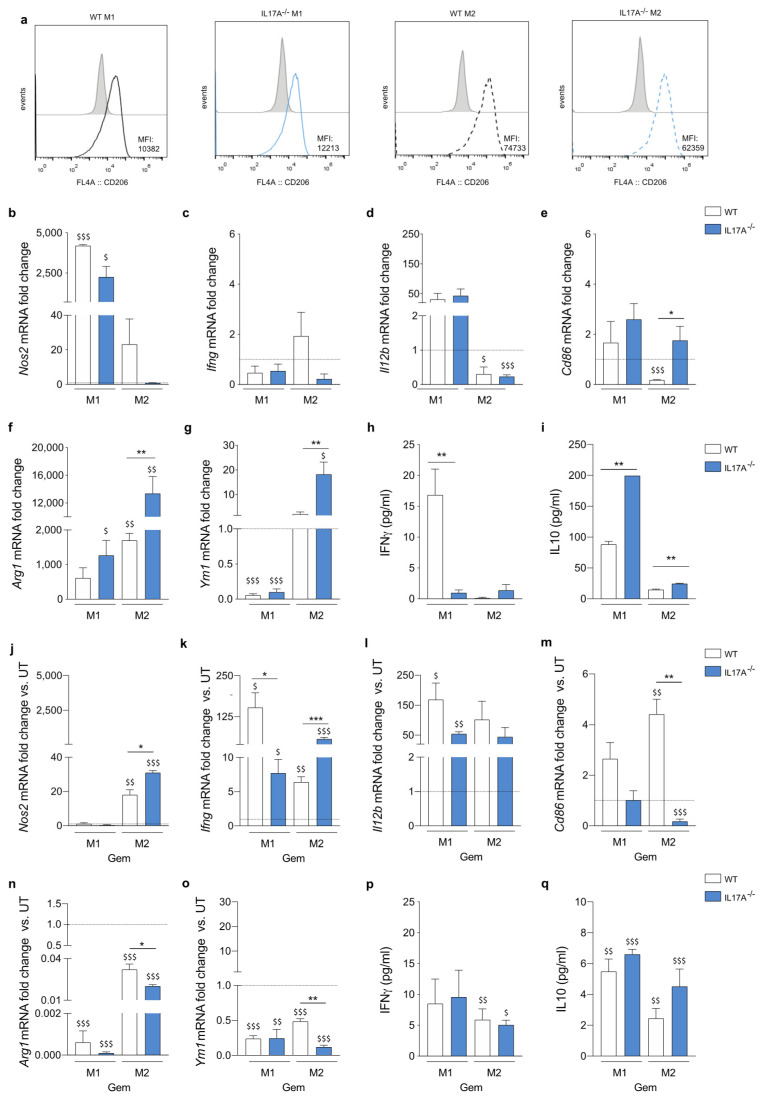
The absence of IL17A induces unique features in both M1- and M2-like macrophages and renders them differently responsive to gemcitabine. Flow Cytometry (FACS) analysis of fluorescence intensity for Mannose Receptor C type 1 (CD206) (**a**) on wild-type (WT) and Interleukin (IL)17A^−/−^ bone marrow derived macrophages (BMDM) polarized toward the M1 or M2 phenotype. *Nos2*, *Ifng*, *Il12b*, *Cd86*, *Arg1* and *Ym1* mRNA levels in BMDM obtained from WT (white bars) or IL17A^−/−^ mice (blue bars). Relative untreated M0 cells were used as reference for evaluating the fold-change (**b**–**g**). interferon (IFN)γ (**h**) and IL10 (**i**) were quantified by enzyme-linked immunosorbent assay (ELISA) in the supernatants of BMDM derived from WT (white bars) or IL17A^−/−^ mice (blue bars). WT and IL17A^−/−^ BMDM polarized M1- and M2-like cells were untreated or treated with gemcitabine for 24 h and analyzed for the expression of *Nos2*, *Ifng*, *Cd86*, *Il12b*, *Arg1* and *Ym1*. Relative untreated M1- or M2-like cells were used as a reference for evaluating the fold-change (**j**–**o**). IFNγ (**p**) and IL10 (**q**) were quantified by ELISA in the supernatants of BMDM derived from WT (white bars) or IL17A^−/−^ mice (blue bars) treated with gemcitabine. Data are represented as means ± SEM (means ± standard error) of biological replicates. * *p* ≤ 0.05, ** *p* ≤ 0.01, *** *p* ≤ 0.001 values from IL17A^−/−^ macrophages were significantly different from those of corresponding WT cells; ^$^ *p* ≤ 0.05, ^$$^ *p* ≤ 0.01, ^$$$^ *p* ≤ 0.001 values from gemcitabine-treated macrophages were significantly different from those of M1- or M2-like untreated cells.

Macrophages were evaluated for the protein and/or transcript expression of markers typically associated with the M1-phenotype, namely mannose receptor C type 1 (MRC1/CD206), inducible nitric oxide synthase (iNOS), Interferon gamma (IFNγ), CD86, and Interleukin (IL)12b, or markers associated with the M2-phenotype, namely Arginase (ARG)1 and Ym1 (Figure 2a–g).

LPS and M-CSF + IL4 stimuli polarized WT and IL17A^−/−^ BMDM with few differences, already evident in M0 cells. IL17A^−/−^ M0 macrophages, indeed, displayed the tendency to increase *Nos2* and *Ifng* transcripts, and expressed significantly less *Arg1* and *Ym1* than WT M0 cells (Supplementary Figure S1a–f). No significant differences in polarization were observed in the M1-like cells. By contrast, IL17A^−/−^ M2-like cells expressed less CD206, commonly defined as a marker of pro-tumorigenic or “alternatively activated” macrophages, but more *Cd86*, *Arg1* and *Ym1* markers than WT cells (Figure 2a,e–g). We also tested supernatants for the presence of IFNγ and IL10 24 h after polarization. Despite the absence of significant gene induction (Figure 2c), IFNγ was produced by WT M1-like macrophages but not by the IL17A^−/−^ counterparts (Figure 2h). As expected, LPS also elicited IL10 secretion by M1-like cells, but the absence of IL17A further increased its production by both M1- and M2-like macrophages significantly (Figure 2i).

Overall, the absence of IL17A did not impair the in vitro generation of M1-like macrophages, but it induced an atypical M2-like phenotype in which *Cd86* expression reached levels similar to those observed in M1-like macrophages.

### 3.2. IL17A Absence Renders Macrophages Differently Responsive to the Gemcitabine-Induced M2-to-M1-Switch

We previously demonstrated that breast cancer-infiltrating immunosuppressive macrophages released a large amount of IL17A, especially when treated with taxan [11]. Similarly, gemcitabine, a chemotherapy drug with a different mechanism of action from taxan, induced IL17A secretion by both M1- and M2-like in vitro generated cells (Supplementary Figure S1m). We firstly assessed if the tumor-cytotoxic dose of gemcitabine that we used in all the experiments affected macrophage viability. The MTT assay revealed that both WT and IL17A^−/−^ M1- or M2-like macrophages were less active compared to untreated cells, even if they remained viable at 24 h and 48 h after chemotherapy treatment (Supplementary Figure S1n,o). In addition, both IL17A^−/−^ M1- and M2-like untreated macrophages displayed an increased viability compared to WT cells. We assessed the mRNA levels of IL17 receptor family. *Il17ra* transcript was significantly reduced in both WT and IL17A^−/−^ M1- and M2-like macrophages. On the other hand, only minor changes were observed for *Il17rc*, *Il17rd* and *Il17re*, and no *Il17rb* transcript levels were detected (Supplementary Figure S1p, upper panels). Gemcitabine greatly increased the expression of *Il17ra* in WT M1- and M2-like macrophages, but not in IL17A^−/−^ macrophages. In addition, it increased the expression of *Il17rc*, *Il17rd* and *Il17re* in both WT and IL17A^−/−^ M1- and M2-like macrophages. This suggests that M1 and M2 macrophages are more responsive to IL17 family than unstimulated M0 even if IL17A^−/−^ macrophages express lower levels of the IL17RA common chain. Again, *Il17rb* expression was no detectable in both WT and IL17A^−/−^ M1- and M2-like macrophages treated with gemcitabine (Supplementary Figure S1p, lower panels).

Gemcitabine has been demonstrated to induce in vivo and in vitro polarization of macrophages towards an M1-phenotype (Di Caro Gut 2014). We treated macrophages with gemcitabine during their polarization, and analyzed for the expression of genes typically associated with M1- and M2-like phenotypes by qRT-PCR. As expected, gemcitabine induced a significant upregulation of M1-associated markers both in M1- and M2-like cells compared to the relative untreated ones, with differences between WT and IL17A^−/−^ macrophages. *Nos2* and *Ifng* were significantly up-regulated in M2-like cells to a greater extent in the absence of IL17A (Figure 2j,k and Supplementary Figure S1q). However, *Ifng* and *Il12b* were greatly increased in WT M1-like macrophages and to a lesser extent in IL17A^−/−^ M1-like cells (Figure 2k,l). Chemotherapy had opposing effects on *Cd86* expression in M2-like macrophages, as it significantly enhanced *Cd86* expression in WT cells but not in IL17A^−/−^ cells (Figure 2m). Finally, gemcitabine significantly decreased the expression of both *Arg1* and *Ym1* in both WT and IL17A^−/−^ M1- and M2-like macrophages, again to a greater extent in IL17A^−/−^ M2-like cells (Figure 2n,o and Supplementary Figure S1q).

Gemcitabine reduced IFNγ secretion by WT cells, but caused enhanced IFNγ secretion by IL17A^−/−^ M1-like cells (Figure 2p). Notably, gemcitabine significantly enhanced IFNγ production by both WT and IL17A^−/−^ M2-like cells, confirming the up-regulation of gene transcripts (Figure 2p). Conversely, gemcitabine significantly reduced IL10 secretion by both WT and IL17A^−/−^ M1-like and M2-like macrophages (Figure 2q).

In conclusion, gemcitabine enhanced the expression of M1-associated genes in both M1- and M2-like macrophages, and simultaneously reduced those associated with the M2 phenotype to a greater extent in the absence of IL17A (Supplementary Table S1).

### 3.3. The Absence of IL17A Attenuates Metabolic Fluxes of M1- and Particularly M2-Like Macrophages 

Since phenotype is a direct reflection of intracellular metabolism [20], we investigated if the absence of IL17A could rewire the metabolic demand of macrophages. 

We represented the raw data of each metabolic pathway as heat maps. Color codes represent values evaluated in each WT and IL17A^−/−^ macrophage populations. We considered values obtained from untreated (UT) M0 cells from each genotype as the basal condition (black values) to set the relative color scale.

**Figure 3:**
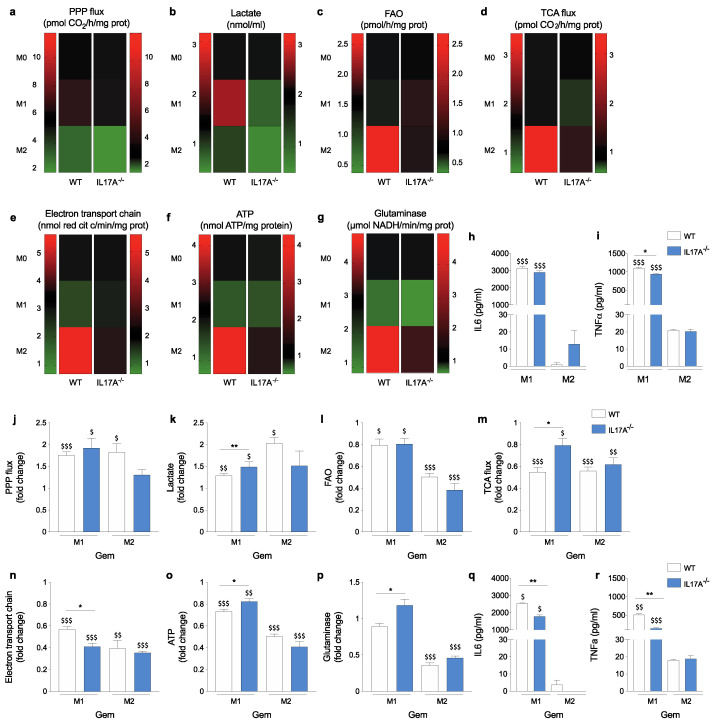
The absence of IL17A attenuates metabolic fluxes of macrophages and differently modulates gemcitabine-induced metabolic changes. WT- and IL17A^−/−^-derived BMDM were polarized toward the M1 or M2 phenotype and analyzed for metabolic activities. Heatmaps represents values evaluated in each WT and IL17A^−/−^ macrophage population. Unpolarized (M0) cells were used as baseline, and are represented as black boxes. Analysis of [1-^14^C] glucose flux through the Pentose Phosphate Pathway (PPP) assessed by ^14^CO_2_ release (**a**); quantification of lactate levels (**b**); analysis of fatty acid beta oxidation (FAO) expressed as amount of [1-^14^C] palmitic acid metabolized to produce ^14^CO_2_ (**c**); analysis of oxidative phosphorylation measured as Tricarboxylic Acid (TCA) cycle rate, evaluated by measuring CO_2_ emission after radiolabeling cells with [1-^14^C] acetyl-coenzyme A (**d**); analysis of mitochondria electron transport chain activity (**e**) and ATP production (**f**), analysis of glutaminase activity (**g**). IL6 (**h**) and TNFα (**i**) production was quantified by ELISA in supernatants from WT- and IL17A^−/−^ M1- or M2-like macrophages (white and blue bars, respectively). WT- and IL17A^−/−^ BMDM (white and blue bars, respectively) were polarized toward the M1 or M2 phenotype and untreated (UT) or treated for 24 h with gemcitabine. Bar graphs represent the fold-change calculated for gemcitabine-treated M1- or M2-like macrophages versus corresponding UT samples: PPP (**j**), lactate production (**k**), FAO (**l**), TCA flux (**m**), Electron Transport Chain (ETC) (**n**), ATP production (**o**) and glutaminase activity (**p**). IL6 (**q**) and TNFα (**r**) production was quantified by ELISA in supernatants from WT- and IL17A^−/−^ M1- or M2-like macrophages treated with gemcitabine for 24 h. Data are represented as means ± SEM of biological replicates. * *p* ≤ 0.05, ** *p* ≤ 0.01, values from IL17A^−/−^ macrophages were significantly different from those of corresponding WT cells; ^$^ *p* ≤ 0.05, ^$$^ *p* ≤ 0.01, ^$$$^ *p* ≤ 0.001 values from gemcitabine-treated macrophages were significantly different from those of untreated M1- or M2-like cells.

The PPP and glycolysis are two of the first utilizers of glucose, and are key metabolic events in M1-like macrophages [21]. In IL17A^−/−^ M0 cells, the basal PPP rate was lower than that of WT cells (3.13 ± 0.2 vs. 5.61 ± 0.19; *p* = 0.0009) (Figure 3a), while the lactate production was higher than in WT cells (2.05 ± 0.04 vs. 1.38 ± 0.15; *p* = 0.0142) (Figure 3b). WT M1-like cells displayed a trend of an increase in PPP activity (6.67 ± 0.64 vs. 5.61 ± 0.19), and—more significantly—in lactate production (2.43 ± 0.15 vs. 1.38 ± 0.15; *p* = 0.0079), one of the by-products of glycolysis (Figure 3a,b). By contrast, the PPP did not change in IL17A^−/−^ M1-like cells (3.54 ± 0.19 vs. 3.13 ± 0.2) whereas lactate production was significantly reduced (0.97 ± 0.02 vs. 2.05 ± 0.04; *p* = 0.0001) (Figure 3b).

As expected, both WT and IL17A^−/−^ M2-like macrophages displayed a significant reduction of the PPP (3.06 ± 0.18 vs. 5.61 ± 0.19; *p* = 0.006 and 1.99 ± 0.11 vs. 3.13 ± 0.2; *p* = 0.0079 respectively) and glycolysis (0.96 ± 0.06 vs. 1.38 ± 0.15 *p* = 0.062 and 0.49 ± 0.04 vs. 2.05 ± 0.04; *p* < 0.0001 respectively).

In addition, pro-inflammatory M1-like macrophages usually display low activity of the tricarboxylic acid cycle (TCA), fatty acid oxidation (FAO) and glutaminolysis, as they employ glycolysis and the PPP to meet their ATP demand. The opposite is mainly true for M2 macrophages that obtain ATP through active oxidative phosphorylation (OXPHOS) [12]. Indeed, FAO and TCA were lower and unchanged in WT M1-like cells compared to untreated M0 respectively (1.31 ± 0.03 vs. 1.48 ± 0.09 and 1.58 ± 0.1 vs. 1.57 ± 0.16), while electron transport chain (ETC) activity (2.1 ± 0.03 vs. 3.44 ± 0.1). ATP production (1.36 ± 0.04 vs. 2.33 ± 0.13) and glutaminolysis (1.28 ± 0.04 vs. 2.88 ± 0.12) were significantly down-modulated (*p* = 0.0001 for all). Conversely, all these pathways were greatly increased in WT M2-like macrophages compared to UT M0 cells (FAO: 2.68 ± 0.06 vs. 1.48 ± 0.09, TCA: 3.4 ± 0.09 vs. 1.57 ± 0.16, ETC: 5.61 ± 0.23 vs. 3.44 ± 0.1, ATP: 4.27 ± 0.06 vs. 2.33 ± 0.13, glutaminolysis: 4.86 ± 0.08 vs. 2.88 ± 0.12; *p* < 0.002 for all) (Figure 3d–f heat map). 

In the absence of IL17A, M0 cells displayed lower basal levels of all these pathways compared to WT M0 cells (FAO: 0.72 ± 0.05 vs. 1.48 ± 0.09, TCA: 0.91 ± 0.14 vs. 1.57 ± 0.16, ETC: 1.96 ± 0.05 vs. 3.44 ± 0.1, ATP: 1.06 ± 0.05 vs. 2.33 ± 0.13, glutaminolysis: 1.23 ± 0.07 vs. 2.88 ± 0.12; *p* values < 0.05 for all). However, in IL17A^−/−^ cells, FAO was enhanced in M2-like macrophages (0.95 ± 0.05) but, interestingly, much more in M1-like cells (1.02 ± 0.04; more than 30% and 40%, respectively). Levels of TCA (1.35 ± 0.5), ETC (2.42 ± 0.12) and ATP (1.55 ± 0.11) were less increased in M2-like macrophages compared to that observed in WT M2-like cells (50% vs. 120%, 20% vs. 63% and 46% vs. 90%, respectively) (Figure 3c–f heat maps). Lastly, glutaminolysis was increased in IL17A^−/−^ M2-like (2.03 ± 0.06) similarly to that evaluated in WT cells (Figure 3g heat maps).

We also evaluated the presence of IL6 and TNFα in the supernatants from WT and IL17A^−/−^ polarized macrophages, as it has been demonstrated that both cytokines correlated with an increased glycolysis and PPP activity [12]. Indeed, M1-like macrophages released both IL16 and TNFα to a much greater extent compared to M2-like cells, regardless of the presence or absence of IL17A (Figure 3h,i). This is in line with what is known in literature about the regulation of pro-inflammatory cytokine and TNFα production by glycolytic enzymes. Indeed, glyceraldehyde 3-phospate dehydrogenase is known to be a post-transcriptional regulator of TNFα mRNA, which is suppressed when the enzyme is not engaged with the glycolysis. In addition, enolase seems to contribute to the production of TNFα [12].

Overall, IL17A absence not only reduced the basal metabolic rate, but also differently impacted the typical metabolic changes associated with macrophage polarization (e.g., lactate production downregulation and FAO upregulation in M1-like macrophages).

### 3.4. IL17A Absence Differently Modulates Metabolic Changes Induced by Gemcitabine

To highlight differences induced by gemcitabine, we represented fold-changes in the graphs by using untreated M1- or M2-like macrophages of each genotype as a reference. Gemcitabine treatment increased glucose use through the PPP and glycolysis pathways, as they were enhanced in both M1- and M2-like macrophages to a greater extent in the absence of IL17A (Figure 3j,k).

Gemcitabine mostly reduced the M2-associated pathways, namely FAO, TCA flux, ETC, ATP production and glutaminolysis, in both WT and IL17A^−/−^ M1- and M2-like macrophages, but to a lesser extent for TCA and ATP, and to a greater extent for the ETC in IL17A^−/−^ M1-like macrophages compared to WT M1-like cells (Figure 3l–p). In addition, in the absence of IL17A, gemcitabine significantly increased glutaminolysis in M1-like macrophages (Figure 3p). However, gemcitabine did not increase IL6 and TNFα production in M2-like cells while decreasing their release in M1-like cells to a greater extent in the absence of IL17A (Figure 3h,i). This result was quite unexpected as an increase in glycolysis would have correlate with an increase in both cytokines. On the other hand, even ATP did not parallel the increase in PPP and lactate production in M1. Therefore, more mechanisms can be involved in the regulation of cytokine secretion after gemcitabine treatment.

Overall, gemcitabine was confirmed to enhance pro-inflammatory polarization by further up-regulating (PPP and lactate production) and down-regulating (FAO, TCA, ETC, ATP and glutaminolysis) elevated pathways in the M1 and in M2 conditions, respectively. IL17A absence buffered some of those metabolic changes; it allowed a reduced increase of PPP and lactate production in M2-like cells and a reduced decrease of TCA and ATP production in M1-like cells (see also Supplementary Table S1).

### 3.5. IL17A Neutralization Combined with Gemcitabine Shapes Macrophages towards a “Peculiar” M1-Like Phenotype

To obtain a proof-of-concept that treatment with an anti-IL17A mAb reproduces the phenotype and metabolic changes observed in IL17A^−/−^-derived macrophages, we treated WT macrophages with an anti-IL17A blocking antibody (or its isotype control) during the stimulation with polarizing factors. In a second set of experiments, we treated IL17A^−/−^ BMDM with recombinant IL17A (rIL17A) together the polarizing stimuli. We evaluated lactate production and FAO as metabolic pathways, and *Nos2* and *Arg1* transcripts as M1- and M2-associated markers, respectively.

**Figure 4:**
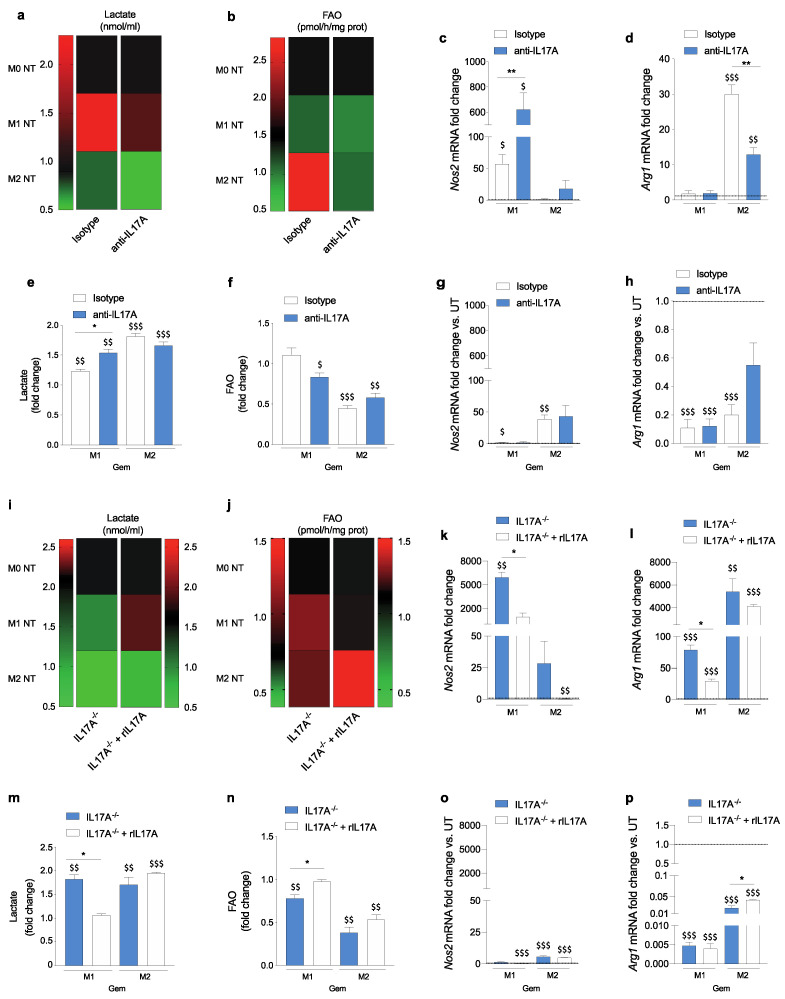
IL17A neutralization combined with gemcitabine shapes macrophages towards a “peculiar” M1-like phenotype. WT M1- and M2-like macrophages were treated with isotype control or anti-IL17A antibody, and either left untreated (heat maps) or treated for 24 h with gemcitabine (bar graphs). Analysis of lactate production (**a**) and Fatty Acid Oxidation (FAO) activity (**b**) relative to the unpolarized M0 macrophages treated with isotype control (first black box of each column). mRNA expression levels of *Nos2* (**c**) and *Arg1* (**d**) are represented as fold-change versus isotype control-M0 cells. White bars represent WT BMDM treated with isotype control antibody while blue bars represent the anti-IL17A-treated BMDM. Lactate production (**e**) FAO activity (**f**) of WT M1-like and M2-like BMDM in the presence of isotype control or anti-IL17A mAb, and treated with gemcitabine. Fold-change was evaluated by considering relative gemcitabine untreated M1- and M2-like cells as references. mRNA expression levels of *Nos2* (**g**) and *Arg1* (**h**) represented as fold-change versus gemcitabine untreated M1- and M2-like cells in the presence of isotype control or anti-IL17A mAb. * *p* ≤ 0.05, ** *p* ≤ 0.01; values from IL17A-Ab treated WT macrophages were significantly different from those of corresponding isotype control-treated WT cells; ^$^ *p* ≤ 0.05, ^$$^ *p* ≤ 0.01, ^$$$^ *p* ≤ 0.001 values from gemcitabine-treated macrophages were significantly different from those of M1- or M2-like gemcitabine untreated cells. Heat maps represent lactate production (**i**) and FAO activity (**j**) of IL17A^−/−^ BMDM, unstimulated or stimulated with rIL17A. mRNA expression levels of *Nos2* (**k**) and *Arg1* (**l**) expressed as fold-change versus unstimulated M0 cells. White and blue bars represent IL17A^−/−^ BMDM unstimulated or stimulated with rIL17A, respectively. Lactate production (**m**) and FAO activity (**n**) of IL17A^−/−^ M1- and M2-like cells in the presence or absence of rIL17A, and treated with gemcitabine. Fold-change was evaluated by considering relative M1- and M2-like cells not treated with gemcitabine as references. White and blue bars represent IL17A^−/−^ BMDM unstimulated or stimulated with rIL17A, respectively. mRNA expression levels of *Nos2* (**o**) and *Arg1* (**p**) represented as fold-change versus M1- and M2-like IL17A^−/−^ macrophages not treated with gemcitabine. All data are represented as means ± SEM of biological replicates. * *p* ≤ 0.05, values from rIL17A-treated IL17A^−/−^ macrophages were significantly different from those of unstimulated IL17A^−/−^ cells; ^$$^ *p* ≤ 0.01, ^$$$^ *p* ≤ 0.001 values from gemcitabine-treated macrophages were significantly different from those of M1- or M2-like untreated cells.

Adding the anti-IL17A neutralizing mAb decreased lactate production in both M1 (1.32 ± 0.06 vs. 2.25 ± 0.14) and M2 (0.535 ± 0.095 vs. 0.73 ± 0.04) conditions, and decreased FAO in M2-like macrophages (1.055 ± 0.115 vs. 2.83 ± 0.08) compared to control M1- and M2-like cells (Figure 4a,b), similarly to that observed in IL17A^−/−^ cells (Figure 3b,c). Unlike what was observed in IL17A^−/−^ macrophages, IL17A pharmacological neutralization significantly increased *Nos2* and decreased *Arg1* transcription in M1- and in M2-like cells, respectively (Figure 4c,d). 

Gemcitabine treatment promoted lactate production and reduced the FAO rate by both M1- and M2-like cells, and to a greater extent in M1-like cells treated with anti-IL17A mAb (Figure 4e,f). Gemcitabine treatment increased *Nos2* and decreased *Arg1* mRNA levels in M2-like cells condition regardless of the presence of anti-IL17A (Figure 4g,h). These results confirmed that gemcitabine favored the shift towards an M1 phenotype, and that IL17A neutralization mostly affected metabolic changes.

Of note, adding rIL17A to IL17A^−/−^ BMDM restored lactate production (1.9 ± 0.03 vs. 1.205 ± 0.045) and FAO activity (1.475 ± 0.045 vs. 1.005 ± 0.115) in M1- and M2-like macrophages, respectively, compared to the untreated cells (Figure 4i,j). Adding rIL17A decreased *Nos2* and *Arg1* transcript levels in both M1- and M2-like IL17A^−/−^ cells, and to a greater extent in M1-like macrophages (Figure 4k,l).

Gemcitabine treatment increased lactate production and decreased the FAO rate in M1- and M2-like macrophages to a similar extent as that of IL17A^−/−^ cells (Figure 4m,n). Adding rIL17A restored these pathways to levels similar to those evaluated in WT BMDM (Figure 4m,n). Similarly, the presence of rIL17A during gemcitabine treatment restored *Nos2* and *Arg1* mRNA levels to a similar extent to that observed in WT polarized macrophages (Figure 4n,p).

Overall, the pharmacological neutralization of IL17A mostly modulated the metabolism of gemcitabine-treated polarized macrophages, similar to that observed in IL17A^−/−^ cells. Likely, adding the rIL17A to gemcitabine-treated macrophages rescued the wild-type phenotype and behavior.

### 3.6. IL17A Depletion Increases the Phagocytosis Rate in Macrophages

**Figure 5:**
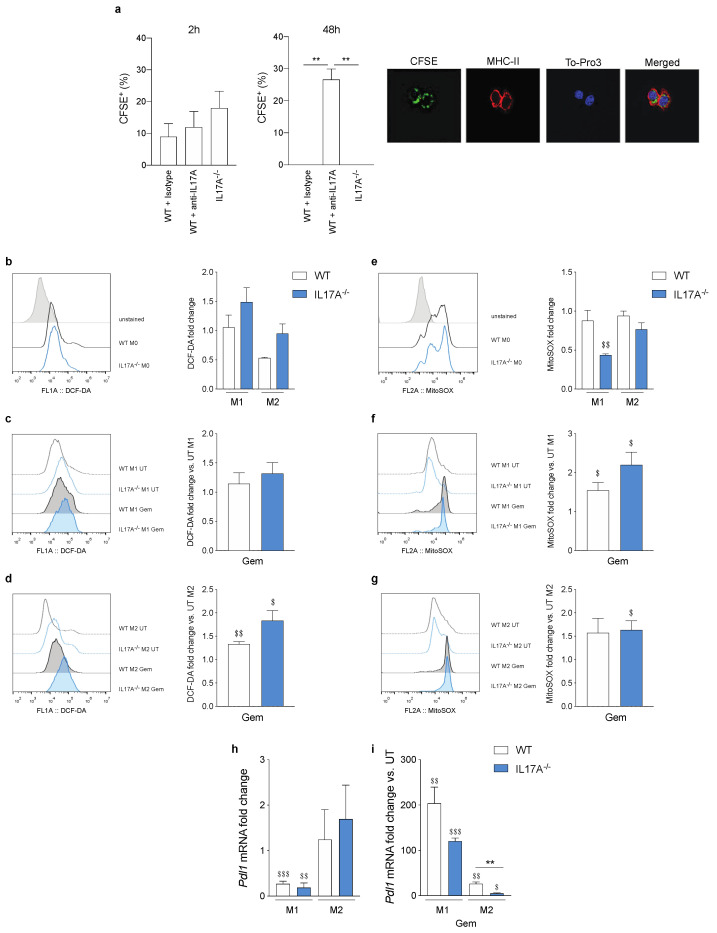
IL17A depletion increases the phagocytosis rate in macrophages. Bar graphs represent the percentage of Carboxyfluorescein Succinimidyl Ester^+^ (CFSE) BMDM after 2 h and 48 h of co-culture with CFSE-stained/gemcitabine-treated PDAC cells (confocal microscopy representative pictures on the right) (**a**). ** *p* ≤ 0.01 values from IL17A mAb-treated WT macrophages were significantly different from those of corresponding WT cells treated with Ig isotype control. FACS analyses of 2′,7′-Dichlorofluorescin diacetate (DCF-DA^+^) (**b**–**d**) and MitoSOX^+^ (**e**–**g**) in untreated M0, M1- and M2-like WT or IL17A^−/−^ BMDM (histograms) or those treated with gemcitabine (white and blue bars, respectively). ^$^ *p* ≤ 0.05, ^$$^ *p* ≤ 0.01 values from gemcitabine-treated macrophages were significantly different from those of M1- or M2-like untreated cells. mRNA expression levels of *Pdl1* detected in WT (white bars) or IL17A^−/−^ (blue bars) in M1- and M2-like macrophages that were untreated or treated with gemcitabine (**h**,**i**). Fold-changes were calculated versus M0 (**h**) or M1- and M2-like untreated (**i**) macrophages. Data are represented as means ± SEM of biological replicates. ** *p* ≤ 0.01 values from IL17A^−/−^ macrophages were significantly different from those of corresponding WT cells; ^$^ *p* ≤ 0.05, ^$$^ *p* ≤ 0.01, ^$$$^ *p* ≤ 0.001 values from gemcitabine-treated macrophages were significantly different from those of M1- or M2-like untreated cells.

To understand how the metabolic status may influence the functional activity of macrophages, and particularly their ability to take-up damaged cells, we tested the phagocytosis of gemcitabine-treated Pancreatic Ductal Adenocarcinoma (PDAC) cells stained with the cell-tracking CFSE in WT and IL17A^−/−^-derived cells. We analyzed the percentage of CFSE-positive macrophages with a confocal microscope at an early (2 h) and a late time point (48 h) from the co-culture with CSFE-stained tumor cells. IL17A^−/−^ and anti-IL17A mAb-treated WT BMDM showed a slight increase in CFSE positivity compared to control WT cells, already at 2 h after pulsing (Figure 5a). Interestingly, 48 h after pulsing, only anti-IL17A mAb-treated WT BMDM still showed the ability to take up CSFE^+^ tumor cells (Figure 5a).

Another macrophage function affected by metabolism and related to phagocytosis is ROS production. We stained WT and IL17A^−/−^ BMDM with DCF-DA and MitoSOX to evaluate the presence of cytosolic and mitochondrial ROS, respectively. Both M0 macrophages showed similar levels of cytosolic and mitochondrial ROS (Figure 5b,e histogram). However, the absence of IL17A caused M1- and M2-like macrophages to specifically accumulate much more cytosolic ROS and less mitochondrial ROS than the corresponding WT cells (Figure 5b,e graphs), confirming the hypothesis of higher FAO and OXPHOS activity in IL17A^−/−^ cells. Gemcitabine increased the production of both cytosolic and mitochondrial ROS in all cells to a greater extent than cytosolic and mitochondrial ROS in IL17A^−/−^ M2- and M1-like cells (Figure 5c,d,f,g graphs).

In a previous study, we demonstrated that taxan-induced ROS increased PD-L1 expression in BMDM and isolated tumor-associated macrophages (TAM) from breast tumors [11]. Here, we investigated *Pdl1* gene expression in WT and IL17A^−/−^ polarized macrophages. As expected, both WT and IL17A^−/−^ M2-like macrophages showed higher levels of *Pdl1* compared to M1-like cells (Figure 5h), but gemcitabine treatment significantly increased *Pdl1* expression in WT M1-like macrophages compared to M2-like cells by 10-fold (Figure 5i). Of note, the absence of IL17A impaired this marked increase of the *Pdl1* transcript in gemcitabine-treated macrophages (Figure 5i).

### 3.7. IL17A Depletion Decreases T Regulatory Cells Infiltrating Pancreatic Cancer

**Figure 6:**
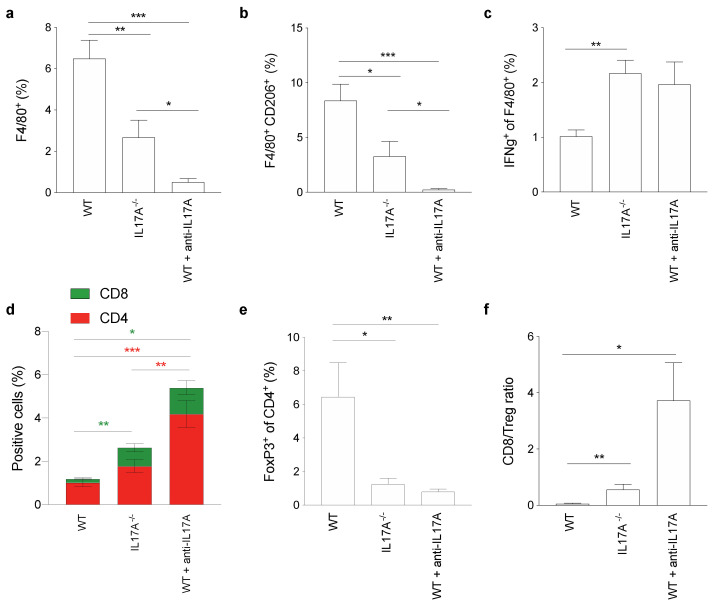
IL17A depletion decreases T regulatory cells infiltrating pancreatic cancer. FACS analyses of dissociated Formalin-Fixed Paraffin-Embedded (FFPE) tumor masses of WT (untreated or treated with anti-IL17A) and IL17A^−/−^ mice orthotopically injected with PDAC cells. Bar graphs represent the percentage of F4/80^+^ (**a**), F4/80^+^ CD206^+^ (**b**), IFNγ^+^ gated on the F4/80^+^ cells (**c**), CD4^+^ and CD8^+^ (**d**), FoxP3^+^ gated among the CD4^+^ cells (**e**) and the CD8/Treg ratio (**f**) tumor-infiltrating immune cells. Data are represented as means + SEM of biological replicates. * *p* ≤ 0.05, ** *p* ≤ 0.01, *** *p* ≤ 0.001 values from IL17A^−/−^ or anti-IL17A mAb-treated WT mice were significantly different from those in WT mice.

To assess if the phenotype and functional status of macrophages (differentiated in the absence of IL17A) paralleled a different tumor microenvironment (TME) in vivo, we took advantage of having FFPE pancreatic tumors excised from WT mice orthotopically injected with PDAC cells, and untreated or treated with the anti-IL17A mAb. At the same time, we also analyzed PDAC samples from IL17A^−/−^ mice orthotopically injected with syngeneic cells. After mechanical and enzymatical dissociation, samples were stained and analyzed by flow cytometry (Supplementary Figure S2). Notably, there was a reduction of F4/80^+^ tumor-associated macrophages (TAM) and especially in F4/80^+^ CD206^+^ cells in tumors from IL17A^−/−^ mice and with a greater extent in tumors from WT mice treated with the anti-IL17A mAb (Figure 6a). TAMs were decreased in the absence of IL17A and this decrease was even greater in mice receiving the anti-IL17A mAb (Figure 6b). Conversely, the percentage of F4/80^+^ TAMs producing IFNγ was almost doubled in the absence of IL17A (Figure 6c).

We also observed a significant increase of CD8^+^ T-cells in mice depleted of IL17A, both genetically and pharmacologically. Similarly, CD4^+^ T-cells were increased in the absence of IL17A, although a statistically significant difference was only observed in pharmacologically IL17A-depleted mice (Figure 6d). Regulatory T-cells (Tregs), instead, were significantly reduced in both genetically and pharmacologically IL17A-depleted mice (Figure 6e); indeed, the CD8/Treg ratio was significantly increased in the absence of IL17A (Figure 6f).

In conclusion, the unique metabolic phenotype displayed by macrophages in the absence of IL17A suggests their ability to recruit more effector T-cells into the tumor area, and also potentially create an inflammatory microenvironment for antigen-presenting cells that take-up dying tumor cells. Alongside macrophages, we have also demonstrated that IL17A significantly affects cancer-associated fibroblasts by modulating their transcriptional profile and secretome [22]. Indeed, they produced less myeloid/granulocytic recruiting factors and favored a Th1 response. Therefore, the depletion of IL17A may have important consequences in the tumor microenvironment targeting different cell populations.

## 4. Discussion

The present study was designed to characterize the role of IL17A in macrophage polarization and in the response to gemcitabine. IL17A is an interesting pro-inflammatory cytokine that has been recently proposed as a driver in PDAC progression [23,24]. It is mainly produced by Th17 and γδT cells, but it has also been reported to be produced by innate cells where it regulates activation and polarization [25,26]. Gemcitabine is one of the most widely used chemotherapy agents in first- and second-line treatment of PDAC, where the introduction of new chemotherapeutic drugs has led to relatively small benefits. One of the reasons is the highly immunosuppressive TME that characterizes PDAC. TAMs constitute one of the most abundant populations of PDAC, and can derive from the differentiation of monocytes recruited by tumor-produced CCL2 and CCL5, or from embryonic precursors [27]. After differentiation into macrophages, TAMs contribute to forming a typical suppressive milieu [28], although some differences based on their origin have been reported [27]. TAMs can suppress CD8^+^ activity, by secreting TGFβ and IL10, which limit the production of perforin and granzymes, and also induce Tregs. These CD4^+^CD25^+^Foxp3^+^ T cells can, indeed, originate in the thymus or from the conversion of conventional T-cells upon T-cell receptor stimulation in the presence of TGFβ [29]. Recently, Halbrook et al. demonstrated that TAMs increase tumor chemoresistance, and in particular, PDAC resistance to gemcitabine, by releasing pyrimidines such as deoxycytidine, which directly compete with the drug and hinder its efficiency [14]. Of note, released pyrimidine is a feature of alternatively activated macrophages. Therefore, precise regulation of macrophage activation is crucial for controlling tumor progression. The importance of TAMs has been demonstrated by the fact that either their depletion from the TME [27,30] or their activation through the anti-CD40 antagonist [31] or PI3K inhibitor [32] was shown to positively correlate with PDAC growth inhibition and gemcitabine efficacy [14].

Metabolism is crucially important as a key hallmark, even in a tumor context. It has been recently demonstrated that metabolic pathways not only provide energy, but also regulate the phenotype and activities of macrophages. Therefore, metabolic reprogramming could be an elegant way to skew suppressive macrophages towards anti-tumor cells. Different stimuli, not only nutrients, act on macrophage metabolism and hence their “activation status”; among these, are cytokines. We have recently developed a PDAC mouse model lacking the IL17A gene, and we observed a unique TME that efficiently supported the anti-tumor response induced by an anti-tumoral vaccine [22]. Indeed, IL17A, besides the well-known IFNγ, IL4, IL6, IL10 and IL13 cytokines, can directly activate macrophages [25]. Interestingly, IL17A has been described as eliciting atypical M2-like and mixed M1 and M2 phenotypes in macrophages isolated from an inflammatory context, such as atherosclerosis and psoriasis [9,25]. We observed that M0 IL17A^−/−^ macrophages displayed a different phenotype compared to WT counterparts in terms of higher expression levels of *Nos2* and *Ifng*, and lower expression levels of *Arg1* and *Ym1* transcripts. Similar findings were obtained by Nakai et al. in a study regarding the role of IL17A in macrophage activation [25]. They observed that the antibody anti-IL17A increased *Nos2* in macrophages in the skin of B6 mice. In addition, Nishikawa and colleagues observed that mice IL17A^−/−^ were characterized by lower mRNA levels of *Arg1* and other M2-phenotype associated genes in inflamed colon tissues [10]. Here we report that the absence of IL17A per se did not impair in vitro macrophage polarization, but it attenuated the potency of IL4 in skewing macrophages towards an M2-like phenotype and metabolism. It would be very interesting to investigate if IL17A may affect gene transcription through the activation of epigenetic regulators. This deserves a specific study. There are many evidences that one of the downstream cytokine effects is modulating epigenetic regulators to determine plasticity and functions of immune cells. Many studies, for example, highlighted how different cytokines contribute to the Th17 plasticity [33], and even our group demonstrated how the cytokines released by TAM influenced epigenetic regulation of *Tbet*, *Il10* and *Pdcd1* promoters in T cells [34].

Gemcitabine has been reported to indirectly affect macrophages in vitro when stimulated with conditioned media from tumor cells pre-treated with chemotherapy. Our results demonstrate a direct effect of gemcitabine on macrophages, which were strongly pushed towards an inflammatory phenotype. These results partly confirmed those reported by Di Caro et al. who described a positive correlation between CD68-TAM and the chemotherapy response [15]. To explain this effect, in vitro experiments revealed the ability of gemcitabine to improve cytotoxic activity as well promoting an M1-like phenotype in macrophages. Indeed, we observed that adding gemcitabine to polarizing stimuli further enhanced the expression of *Ifng*, *Cd86* and *Il12b* genes in M1-like macrophages, but also induced their expression in M2-like macrophages, where gemcitabine strongly inhibited the expression of *Arg1* and *Ym1* genes. As a consequence, the absence of IL17A cooperated with gemcitabine in reverting M2-like metabolism by buffering the FAO pathway and ATP production through the OXPHOS. This paralleled the significant decrease of *Arg1*, *Ym1* and *Pdl1* and increase in *Ifng* mRNA expression, together with ROS production. PDL1 expression, indeed, has been linked to higher OXPHOS activity induced by IL4 in alternatively activated macrophages [12] and by chemotherapy-induced oxidative stress [11].

The different activation status is essential for controlling intruder attacks, anti-tumor responses and maintenance of tissue homeostasis. In this context, the immuno-metabolism recently highlighted differences between macrophage subtypes. Glycolytic enzymes are responsible for HIF-1α stabilization, which, in turn, induces inflammasome activation and IL1b production, STAT3-dependent IL6 secretion, TNFα production and finally, antiviral and antibacterial responses by inflammatory macrophages [12]. M1-like macrophages display a disrupted TCA in two places [35,36], which leads to increased lipogenesis and production of NO [37], which increases stabilization of HIF-1α and production of ROS [38,39]. 

In our study, WT M1-like macrophages displayed increased glycolysis, as supported by the increased production of lactate and enhanced PPP, as expected [12]. In the absence of IL17A M1-like macrophages did not, however, increase lactate production but only PPP, and unexpectedly displayed a higher FAO rate compared to WT cells. Gemcitabine treatment further increased both lactate production and the PPP. In addition, IL17A absence strongly affected the metabolic changes induced by IL14-polarization. Indeed, IL17A^−/−^ M2-like macrophages displayed a slight increase of FAO, TCA, ETC, ATP production and glutaminolysis compared to WT cells [12]. Of note, a lower production of lactate and an increase in the FAO rate were also observed in WT-derived macrophages treated with the anti-IL17A mAb. These results suggest that, in the absence of IL17A, macrophages are not glucose “greedy” like WT cells. Therefore, in the TME, macrophages would not participate in increasing acidification, and would favor myeloid-derived suppressor cell induction and cytotoxic CD8 T and NK cell activity impairment [40,41]. Indeed, glucose consumption by tumors and macrophages metabolically impairs T cells, leading to their reduced mTOR activity, glycolytic capacity and IFN-γ production, thereby allowing tumor progression [42]. Inhibition of glycolysis in TAM has also been reported to be sufficient in limiting their pro-angiogenic function and the induction of the epithelial-to-mesenchymal transition in tumor cells [43].

Another well-known mechanism for hindering the T-cell response is the upregulation of immune-checkpoint molecules. As previously reported, gemcitabine increases the expression of *Pdl1* [44], which appears to be related to higher OXPHOS activity associated with IL4 stimulation [12]. Here, we observed that the absence of IL17A significantly reversed this phenomenon, and M2-like macrophages treated with gemcitabine expressed 5-fold less *Pdl1* than the WT counterpart, despite the accumulation of cytosolic ROS that support higher OXPHOS rate. M1-like macrophages treated with gemcitabine also increased their *Pdl1* expression, but again, in the absence of IL17A, the *Pdl1* transcript was decreased by 50%. It would be interesting to investigate if this effect in the absence of IL17A is due to ROS-dependent activation of Nuclear Factor Kappa B (NFkB), as for taxan [11].

Originally FAS and FAO were thought to fuel OXPHOS and, therefore, an anti-inflammatory phenotype. The absence of fatty acid transporter protein 1 (FATP1) was, indeed, reported to increase glycolysis, which paralleled an increased *Nos2* and a decreased *Arg1* expression, without changes in other surface macrophage markers [45]. However, the lack of carnitine palmitoyl transferase (CPT1), which implies the absence of FAO, did not impair the IL4-induced M2 polarization [46], suggesting that the role of FAO in alternative macrophage activation remains a continuing debate. In the absence of IL17A, M2-associated metabolic pathways did not increase at all, or only very slightly, while glycolysis seemed to increase a little. A similar trend was observed in macrophages from mice lacking FATP1 [45]. This could explain the enhanced transcription of *Nos2* and the decreased transcription of *Arg1* observed in the absence of IL17A. Adding gemcitabine decreased all these pathways, both in WT and IL17A^−/−^ M2-like macrophages, again supporting the idea of a shift towards an antitumoral phenotype. The higher expression of *Nos2* in the absence of IL17A supports the impairment of the ETC and FAO, probably due to the increase in NO production, which impairs the mitochondrial ETC [47]. Of note, adding recombinant IL17A to IL17A^−/−^-derived macrophages restored lactate production and FAO activity in M1- and in M2-like macrophages, which were further increased and decreased by gemcitabine treatment, respectively.

## 5. Conclusions

**Figure 7:**
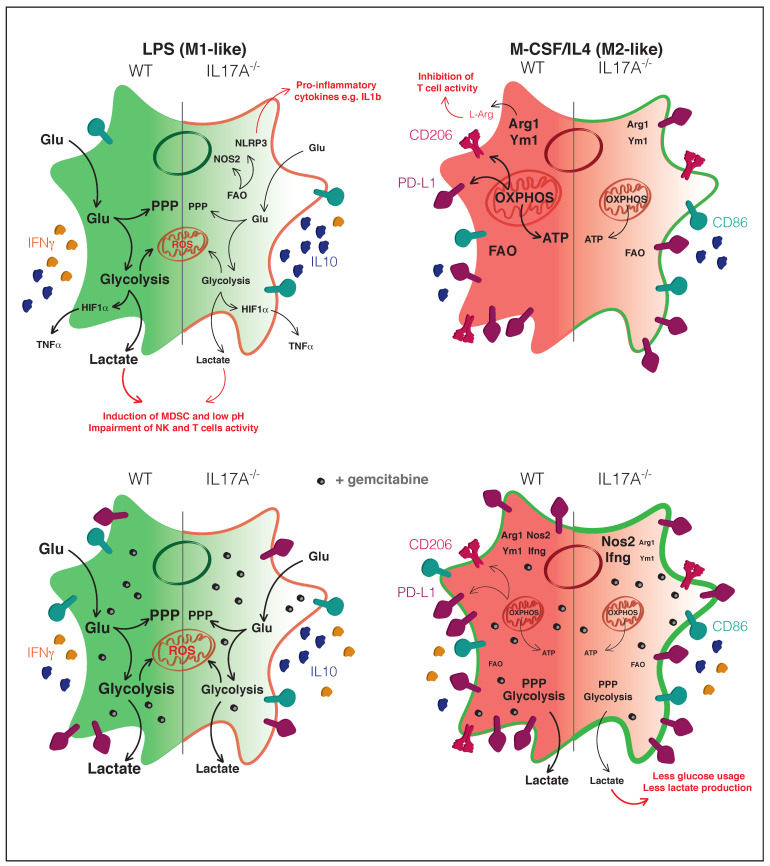
Schematic representation of the IL17A effects on macrophages in the presence or not of gemcitabine.

Overall, our results unveil a novel function of IL17A in modulating macrophage metabolism by attenuating typical M1 and M2 metabolic pathways as schematically shown in Figure 7. This implies the shift of macrophages towards a subset consuming less glucose and producing less lactate, which may orchestrate a more effective antitumoral response. This occurred even after gemcitabine treatment and, therefore, the combination of an anti-IL17A mAb with gemcitabine to modulate the function and activity of macrophages in the TME could be envisioned.

## Figures and Tables

**Table 1:** List of primers.

Gene	Accession Number	Forward	Reverse
*Gapdh*	14433	CATCACTGCCACCCAGAAGACTG	ATGCCAGTGAGCTTCCCGTTCAG
*Nos2*	18126	CTTTGCCACGGACGAGAC	TCATTGTACTCTGAGGGCTGAC
*Ifng*	15978	ATCTGGAGGAACTGGCAAAA	TTCAAGACTTCAAAGAGTCTGAGGTA
*Cd86*	12524	GAAGCCGAATCAGCCTAGC	CAGCGTTACTATCCCGCTCT
*Il12b*	16160	AAGGAACAGTGGGTGTCCAG	GTTAGCTTCTGAGGACACATCTTG
*Arg1*	11846	GAATCTGCATGGGCAACC	GAATCCTGGTACATCTGGGAAC
*Ym1*	12655	AAGAACACTGAGCTAAAAACTCTCCT	GAGACCATGGCACTGAAC G

## Supplementary Materials

The following are available online at https://www.mdpi.com/2076-3921/10/3/422/s1, Figure S1: IL17A affects macrophage polarization, viability and receptor expression, Figure S2: Gating strategy for immune cells infiltrating pancreatic cancer. Table S1: Summary of changes induced by gemcitabine treatment in macrophages in the presence or absence of IL17A.

## Abbreviations

BMDM	Bone Marrow-Derived Macrophages;
CD206	mannose receptor C type 1;
CFSE	Carboxyfluorescein Succinimidyl Ester;
CPT1	Carnitine Palmitoyl Transferase 1;
ETC	Electron Transport Chain;
FAO	Fatty Acid Oxidation;
FAS	Fatty Acid Synthesis;
FATP1	Fatty Acid Transporter Protein 1;
FFPE	Formalin-Fixed and Paraffin-Embedded;
GLS	Glutaminase;
IFNγ	Interferon gamma;
IL	Interleukin;
M-CSF	Macrophage Colony-Stimulating Factor;
NOS	Nitric Oxide Synthase;
OXPHOS	Oxidative Phosphorylation;
PDAC	Pancreatic Ductal Adenocarcinoma;
PPP	Pentose Phosphate Pathway;
ROS	Reactive Oxygen Species;
TAM	Tumor-Associated Macrophages;
TCA	Tricarboxylic Acid;
TME	Tumor Microenvironment.
