# Supplementary material for: IL17A Depletion Affects the Metabolism of Macrophages Treated with Gemcitabine
Source: Antioxidants (Basel). 2021 Mar 10;10(3):422. doi: 10.3390/antiox10030422 (PMC7999796; doi:10.3390/antiox10030422)
Supplement: Supplementary file 1 [file antioxidants-10-00422-s001.pdf]

Supplementary Materials:

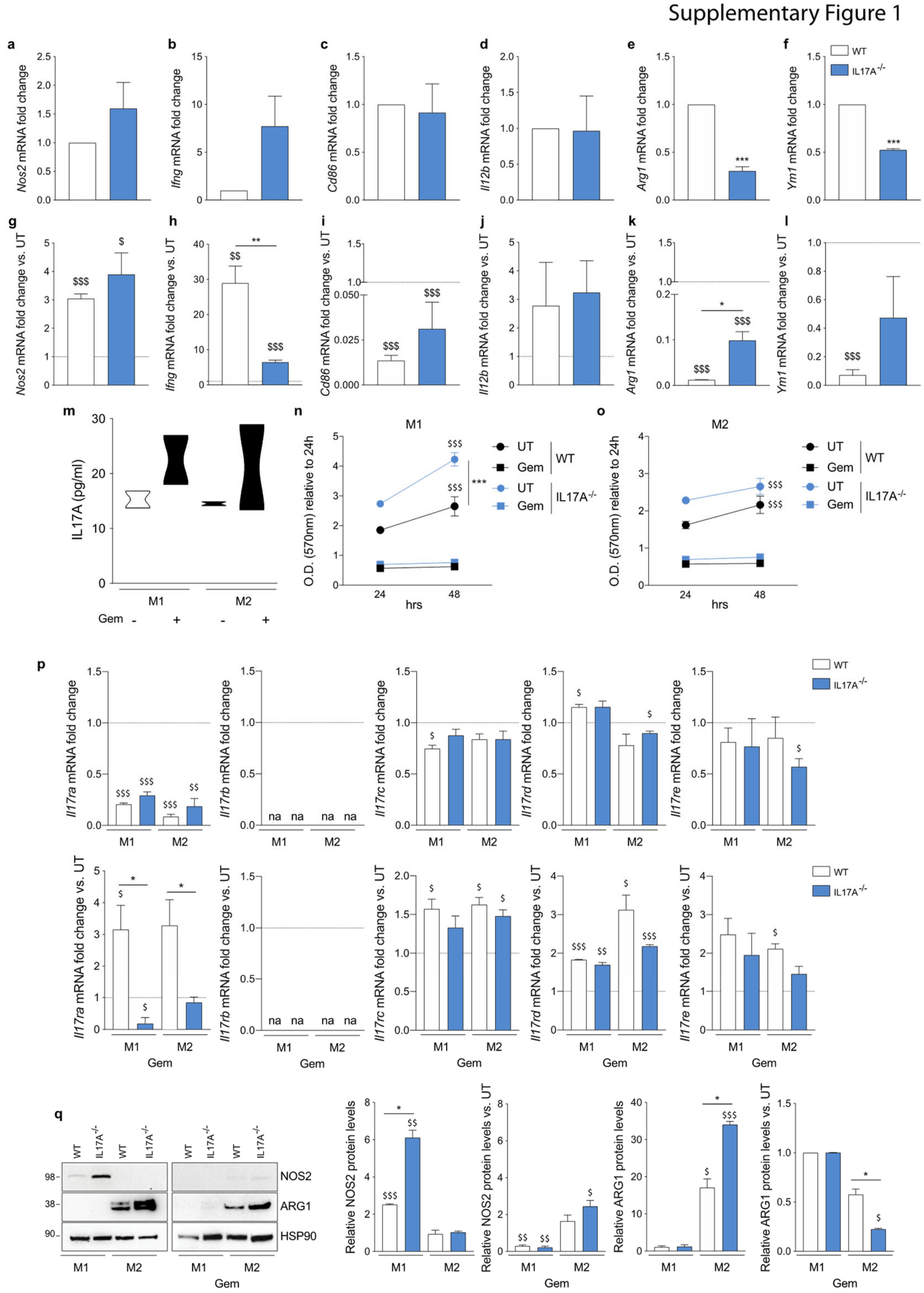

Supplementary Figure S1. IL17A affects macrophage polarization, viability and receptor expression. *Nos2* (**a**), *Ifng* (**b**), *Cd86* (**c**), *Il12b* (**d**), *Arg1* (**e**) and *Ym1* (**f**) mRNA levels in WT (white bars) or IL17A$^{-/-}$ (blue bars) M0 untreated bone-marrow-derived macrophages (BMDMs). ***P ≤ 0.001 values from IL17A$^{-/-}$ macrophages were significantly different from those of WT cells. *Nos2* (**g**), *Ifng* (**h**), *Cd86* (**i**), *Il12b* (**j**), *Arg1* (**k**) and *Ym1* (**l**) mRNA levels in WT (white bars) or IL17A$^{-/-}$ (blue bars) M0 treated with gemcitabine bone-marrow-derived macrophages (BMDMs).*P ≤ 0.05, **P ≤ 0.01 values from IL17A$^{-/-}$ macrophages were significantly different from those of WT cells. *Nos2* (**g**), *Ifng* (**h**), *Cd86* (**i**), *Il12b* (**j**), *Arg1* (**k**) and *Ym1* (**l**) mRNA levels in WT (white bars) or IL17A$^{-/-}$ (blue bars) M0 treated with gemcitabine bone-marrow-derived macrophages (BMDMs).IL17A levels

were quantified by ELISA in supernatants from M1- or M2-like WT BMDMs, untreated or treated with gemcitabine for 24 h (**m**). 24-48 h MTT assay performed with M1(**n**) or M2 (**o**) polarized WT (black symbols) or IL17A$^{-/-}$ (blue symbols) BMDMs. ***P ≤ 0.001 values from IL17A$^{-/-}$ macrophages were significantly different from those of WT cells. $^{\$\$\$}$P ≤ 0.001 values from untreated macrophages were significantly different from those of gemcitabine-treated cells. *Il17ra, Il17rb, Il17rc, Il17rd* and *Il17re* mRNA levels in WT (white bars) or IL17A$^{-/-}$ (blue bars) BMDMs (**p**). Graphs represent the fold-change versus relative untreated M0 cells (upper panels) or M1-like or M2-like cells (lower panels). Data are expressed as means ± SEM of biological replicates. *P ≤ 0.05, **P ≤ 0.01, ***P ≤ 0.001 values from IL17A$^{-/-}$ were significantly different from those in WT macrophages. $^{\$}$P ≤ 0.05, $^{\$\$}$P ≤ 0.01, $^{\$\$\$}$P ≤ 0.001 values from gemcitabine-treated macrophages were significantly different from those of M1-like or M2-like untreated cells. (**q**) Representative Western Blot images for NOS2 and ARG1 with proteins extracted from untreated M1- and M2-like macrophages or those with gemcitabine for 24 h. HSP90 was used as a loading control. Graphs represent the quantification of NOS2 and ARG1 bands from two independent immunoblotting experiments. *P ≤ 0.05 values from IL17A$^{-/-}$ BMDMs were significantly different from those in WT macrophages. $^{\$}$P ≤ 0.05, $^{\$\$}$P ≤ 0.01, $^{\$\$\$}$P ≤ 0.001 values from gemcitabine-treated macrophages were significantly different from those of M1-like or M2-like untreated cells.

Supplementary Table S1. Summary of changes induced by gemcitabine treatment in macrophages in the presence or absence of IL17A.

**Markers and metabolic pathways**		**WT M1**		**IL17A$^{-/-}$ M1**		**WT M2**		**IL17A$^{-/-}$ M2**	
	**Gem**	**-**	**+**	**-**	**+**	**-**	**+**	**-**	**+**
Nos2 (mRNA)									
NOS2 (protein)			nd[a]		nd	nd		nd	
Arg1 (mRNA)									
ARG1 (protein)		nd	nd	nd	nd				
Ifng									
Cd86									
Il12b									
Ym1									
PPP flux									
Lactate									
FAO									
TCA flux									
ETC									
ATP									
Glutaminase									

[a] Not detected by Western Blotting.

Red boxes represent an increase, green boxes a reduction and black boxes unchanged levels compared with control samples: M0 untreated macrophages for all the conditions in the absence of gemcitabine, and M1- or M2-like untreated macrophages when gemcitabine is present.

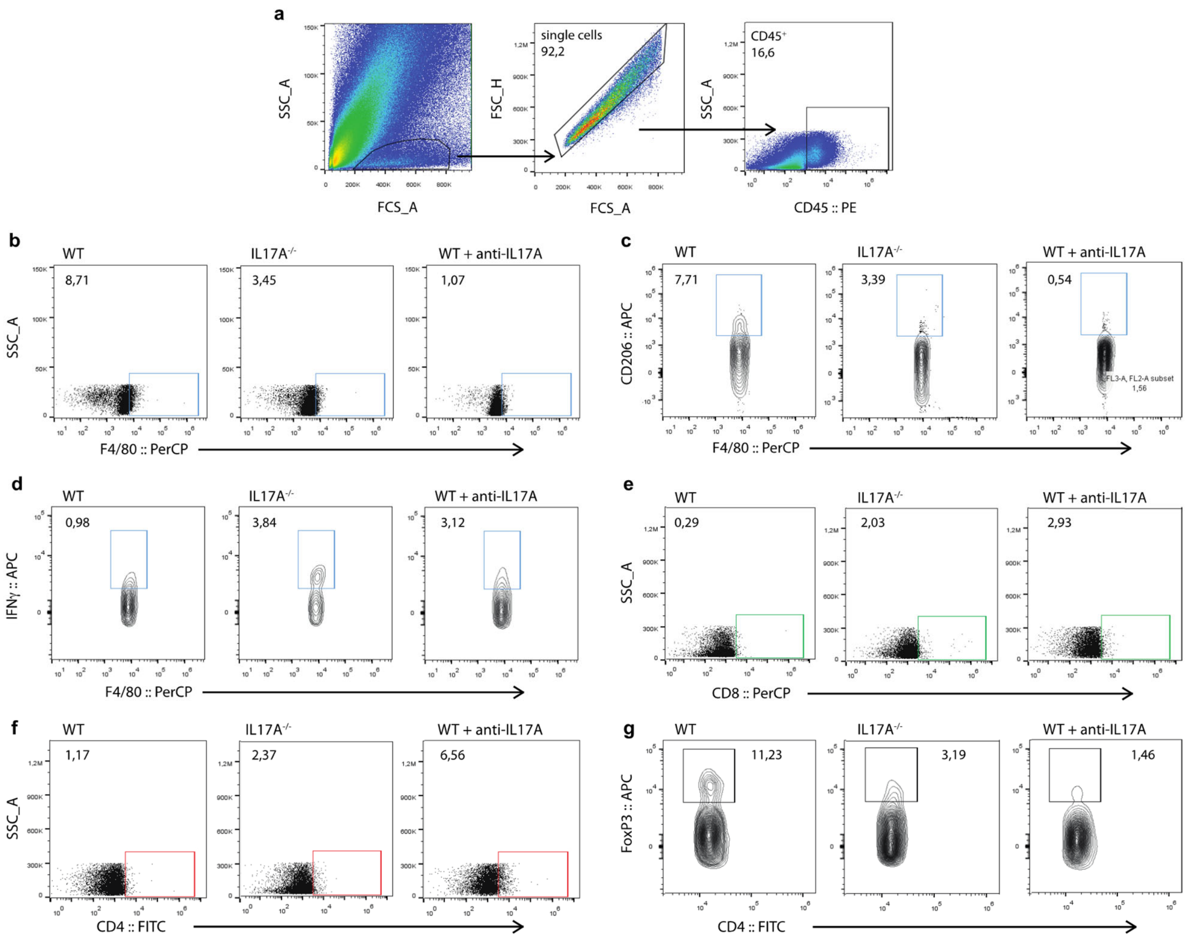

Supplementary Figure S2. Gating strategy for immune cells infiltrating pancreatic cancer. Representative flow cytometry plots for intra-tumor cell populations, showing the gating strategy. Data are representative of n = 5 mice/group. Total $CD45^+$ cells were first gated on single cells deriving from a Forward Scattered (FSC_A)/Side Scattered (SSC_A) plot (**a**). $CD45^+$ cells were further gated for the subsets of interest, namely $F4/80^+$ cells (**b**), $CD206^+$ (**c**) and $IFN\gamma^+$ (**d**) gated on the $F4/80^+$ cells, $CD8^+$ (**e**), $CD4^+$ (**f**) and $FoxP3^+$ gated on the $CD4^+$ cells (**g**).
